# A Hybrid Clustering Method with a Filter Feature Selection for Hyperspectral Image Classification

**DOI:** 10.3390/jimaging8070180

**Published:** 2022-06-28

**Authors:** Junzhe Zhang

**Affiliations:** 1 Department of Atmospheric and Oceanic Sciences, University of California, Los Angeles, CA 90095, USA; zhangjunzhe8868@ucla.edu; 2 College of Resources Science and Technology, Beijing Normal University, Beijing 100875, China

**Keywords:** feature selection, classification, hyperspectral image, similarity measure, K-means

## Abstract

Hyperspectral images (HSI) provide ample spectral information of land cover. The hybrid classification method works well for HSI; however, how to select the suitable similarity measures as kernels with the appropriate weights of hybrid classification for HSI is still under investigation. In this paper, a filter feature selection was designed to select the most representative features based on similarity measures. Then, the weights of applicable similarity measures were computed based on coefficients of variation (CVs) of similarity measures. Implementing the similarity measures as the kernels with weights into the K-means algorithm, a new hybrid changing-weight classification method with a filter feature selection (HCW-SSC) was developed. Standard spectral libraries, operative modular imaging spectrometer (OMIS) airborne HSI, airborne visible/infrared imaging spectrometer (AVIRIS) HSI, and Hyperion satellite HSI were selected to inspect the HCW-SSC method. The results showed that the HCW-SSC method has the highest overall accuracy and kappa coefficient (or F1 score) in all experiments (97.5% and 0.974 for standard spectral libraries, 93.21% and 0.9245 for OMIS, 79.24% and 0.8044 for AVIRIS, and 81.23% and 0.7234 for Hyperion) compared to the classification methods (93.75% and 0.958 for standard spectral libraries, 88.27% and 0.8698 for OMIS, 73.12% and 0.7225 for AVIRIS, and 56.34% and 0.3623 for Hyperion) without feature selection and the machine-learning method (68.27% and 0.6628 for AVIRIS, and 51.21% and 0.4255 for Hyperion). The experimental results demonstrate that the new hybrid method performs more effectively than the traditional hybrid method. This also shed a light on the importance of feature selection in HSI classification.

## 1. Introduction

Remote sensing is a very useful means to monitor and detect land cover/use in a short time frame [1,2,3,4,5]. With the rapid technological development of spectroradiometer and aviation, hyperspectral images (HSI) have been frequently employed to monitor and detect land cover/use change, such as urban mapping, crop health monitoring, and mineral detection [6,7,8]. The primary advantage of HSI is the continuum of the spectrum [9,10,11], which derives continuous spectral curves in the frequency domain or integrated spectral vectors in the reflectance domain.

Classification is a method to differentiate the objects of remote-sensing imagery into different classes, which is an important step to provide a secondary product for mapping, monitoring, and detecting land cover/use change [9,12,13,14,15]. Since HSIs usually have a high spectral and also spatial resolution and the aim of classification of an HSI is to precisely map, monitor, and detect valuable land cover/use change, the classification method of HSIs is different from the classification method for the multispectral image. There are three research topics for the classification method of HSIs: clustering based on graph theory [16,17], clustering by using a machine-learning algorithm [18,19,20], and clustering with the hybrid kernels [21,22,23,24] The clustering based on the graph theory heavily depends on auxiliary space to hold the cache, which needs the graphics processing unit (GPU) and extra random-access memory (RAM). The clustering by using a machine-learning algorithm usually has nonlinear time complexity. When the size of the HSI is large, the performance of this method is slow. A hybrid classification method, which combines two or more similarity measures as the kernels, has the balance of time complexity and space complexity. For example, a dual-clustering-based method was developed to filter the band on HSI [25]. Spectral angle cosine–Euclidean distance (SAC-ED) simply combines two similarity measures [24] The advantage of the hybrid method is that it can analyze the differences in both reflectance and frequency domains. The unknown parts of the hybrid method are the criteria for selecting the kernels and the weights of selected kernels.

Feature selection is a method to find the minimally valuable features that are necessary and sufficient for classification from the raw images [26]. Feature selection can partially or entirely remove the irrelevant or redundant features from images [27]. The aims of feature selection include training classification models faster, reducing the complexity of classification models, improving the accuracy of classification models, and decreasing overfitting. Therefore, feature selection is a good means to provide prior knowledge of HSI and to select the useful similarity measure with the estimated weights for the classification method.

The objective of this paper is to design a feature selection method that can choose the most useful spectral similarity measures with appropriate weights for a hybrid classification method (HCW-SSC) to achieve a better classification result. In Section 2.1, the datasets used in this paper are introduced. In Section 2.2, the workflow of the feature selection and the classification is displayed. In Section 2.3, the indicators used to evaluate the classification results are shown. In Section 2.4, the implementation of this method by using Python is presented. In Section 3, the classification result of this method is compared to the classification methods with no feature selection and machine-learning method. In Section 4, the importance of feature selection in the HCW-SSC classification method and the time complexity of HCW-SSC are discussed. Moreover, the results of the HCW-SSC method from this paper were compared to other papers. In Section 5, the conclusions and contributions of this paper are exhibited. The contribution of this paper is that a hybrid clustering method with a filter feature selection was developed for HSI. The filter feature selection is designed to select the most representative features based on similarity measures and calculate the suitable similarity measures as kernels with the appropriate weights of hybrid classification for hyperspectral imaging.

## 2. Data and Method

### 2.1. Data

Four datasets were employed to test the HCW-SSC method, including standard spectral libraries, operative modular imaging spectrometer (OMIS) (CSA, Shanghai, China) airborne hyperspectral image, airborne visible/infrared imaging spectrometer (AVIRIS) (JPL, Pasadena, CA, USA) hyperspectral image, and Hyperion satellite (JPL, Pasadena, CA, USA) hyperspectral image. The details of the data used in this paper are shown in Table 1. Four preprocessing steps were applied to the HSIs before using them in the classification methods: radiometric calibration was applied; bad and noisy bands were deleted; a minimum noise fraction rotation (MNF) was used to remove the smile/frown effect [28], which causes a significant cross-track and nonlinear disturbances on spectral curves and spectral vectors [29]; and principal component analysis (PCA) was employed to reduce the high-correlated bands [30].

#### 2.1.1. Standard Spectral Libraries

Four standard spectral libraries (Table 2) were used to test the accuracy of the HCW-SSC, including United States Geological Survey (USGS) vegetation and mineral libraries [31], and Chris Elvidge green and dry vegetation libraries [32].

In order to evaluate and validate the accuracy of this method, 80 groups of spectra combinations were selected. For one group of test data, there are three spectral profiles. Two of them belong to the same category and another belongs to a different category based on the description from the spectral library. For example, two spectra of Sporobolus and one spectrum of *Andropogon virginicus* were selected in one group based on the description of USGS vegetation library. The spectra of Sporobolus are similar and different from *Andropogon virginicus*.

#### 2.1.2. OMIS Hyperspectral Image

OMIS is an airborne spectroradiometer, which is developed by the Shanghai Institute of Technical Physics (SITP), Chinese Academy of Sciences (CAS), China [33]. OMIS uses a whiskbroom system to cover the spectral region of visible, infrared, and thermal infrared (400–12,500 nm). It has selectable 64 or 128 bands, 2.8 m spatial resolution, 10 nm spectral resolution, and an IFOV (instantaneous field of view) of 0.003 rad. The OMIS data can be found at http://www.scidb.cn/en (accessed on 8 June 2012).

**Figure 1:**
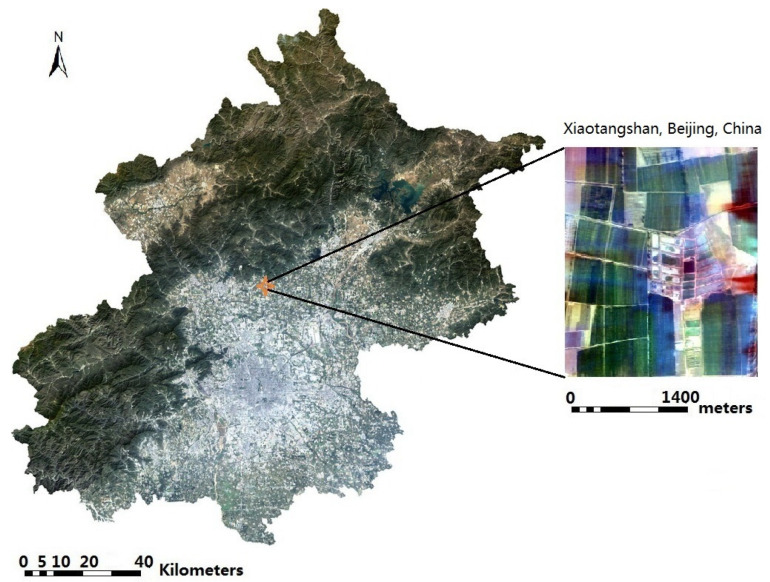
The study area at Xiaotangshan, Beijing, China (OMIS true color synthesis image, R = 699.2 nm, G = 565.4 nm, B = 465.0 nm).

The study area is on Xiaotangshan, Beijing, China. The hyperspectral imagery was obtained on 11 April 2010 (Figure 1). Fifty-one bands between 455.7 nm to 1000.4 nm were selected. The study area is a precision agriculture experimental field, which contains different kinds of wheat [24]. The baseline map was made by field measurement on the experimental field, which was recorded as a shapefile.

#### 2.1.3. AVIRIS Hyperspectral Image

AVIRIS is an airborne spectroradiometer, which is developed by the Jet Propulsion Laboratory (JPL), USA. It has 224 bands ranging from 400 to 2500 nm with a 10 nm bandwidth. AVIRIS is a pushbroom instrument with an 11km-wide swath perpendicular to the satellite motion. The spatial resolution is 20 m [34]. The AVIRIS data can be found at https://aviris.jpl.nasa.gov/dataportal/ (accessed on 9 May 2015).

**Figure 2:**
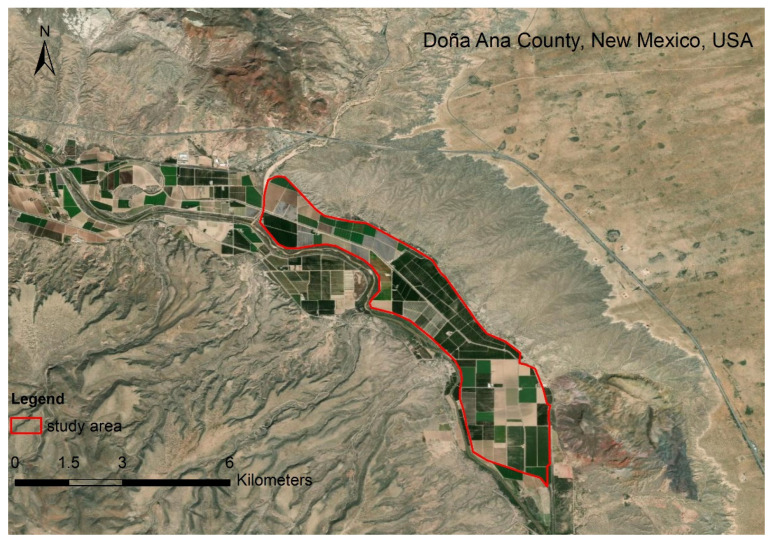
The study area at Doña Ana County, NM, USA.

The study area is in Doña Ana County, NM, USA. The hyperspectral imagery was obtained on 23 May 2011 (Figure 2). One hundred and seventy bands were selected. The study area is in the northern part of San Ysidro city, which contains tree nuts, pepper, grains, and corn [35,36]. The baseline map was made by the high-resolution classification result of the National Agriculture Imagery Program (NAIP) on 25 July 2011. The data can be found at https://nrcs.app.box.com/v/naip (accessed on 9 May 2015).

#### 2.1.4. Hyperion Satellite Hyperspectral Image

Hyperion is a satellite-based spectroradiometer on Earth Observing One (EO-1), which was developed by the National Aeronautics and Space Administration (NASA), USA. It has 242 bands ranging from 357 nm to 2576 nm with a 10 nm bandwidth. Hyperion is a pushbroom instrument with a 7.5 km-wide swath perpendicular to the satellite motion. The spatial resolution is 30 m [37]. The EO-1 data can be found at https://search.earthdata.nasa.gov/search (accessed on 9 February 2015).

**Figure 3:**
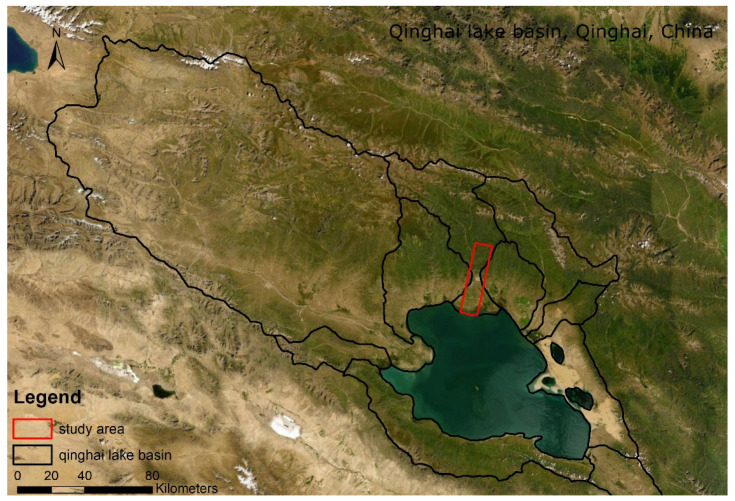
The study area at Qinghai Lake basin, Qinghai Province, China.

The study area is in Qinghai Lake basin, Qinghai province, China. The hyperspectral imagery was obtained on 4 September 2013 (Figure 3). One hundred and seventy-five bands were selected. The study area is in the northern part of the Qinghai Lake basin, which contains winter wheat and rape [38,39]. The baseline map was made by the high-resolution classification result of the Rapideye image on 19 July 2013. The data can be found at https://www.planet.com/products/planet-imagery/ (accessed on 9 May 2015).

### 2.2. Method

**Figure 4:**
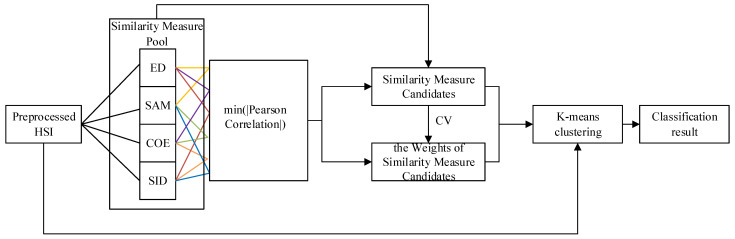
The evaluation scheme for the HCW-SSC method (Euclidean distance (ED), spectral angle cosine (SAC), spectral correlation coefficient (SCC), and spectral information divergence (SID)).

The general workflow has four parts (Figure 4): to extract the features from preprocessed HSI by using Euclidean distance (ED), spectral angle cosine (SAC), spectral correlation coefficient (SCC), and spectral information divergence (SID) similarity measures; to find the minimum absolute Pearson correlation on any of the two (the lines in the same color represents one pair, Figure 4) out of four features; to select the related similarity measures of the features of the minimum absolute Pearson correlation and calculate the coefficients of variation (CVs) of these two similarity measures; and to build a K-means clustering method that contains these similarity measures as the kernels with their weights.

#### 2.2.1. Similarity Measure

**Figure 5:**
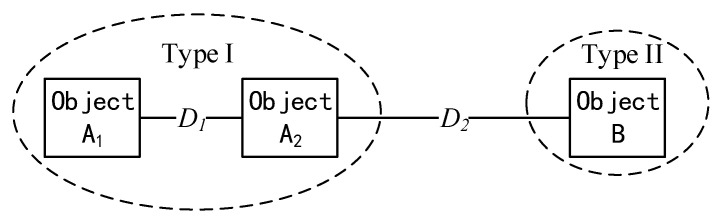
A schematic diagram showing the rule of the similarity measure.

A similarity measure is a function to quantify the similarity between two objects [40]. The rule of similarity measure is “the higher similarity measure value is calculated, the smaller similarity these two objects are”. For example, as shown in Figure 5, D1 (the similarity measure between object A1 and object A2) is smaller than D2 (the similarity measure between object A2 and object B), which means object A1 and object A2 have a high probability to belong to the same category but object B is different from them. The commonly used similarity measures include distance measure (ED) to quantify the difference of brightness between pixel *X_n_* and pixel *X_n_*_+1_ in the frequency domain [41]; consistency measure (SCC) to compare the angle of spectral vectors from pixel *X_n_* and pixel *X_n_*_+1_ in the reflectance domain [42]; dependence measure to measure the difference of shape of spectral curves (SAC) between pixel *X_n_* and pixel *X_n_*_+1_ in the frequency domain [43]; and information measure (SID) to compute the information gain from pixel *X_n_* and pixel *X_n_*_+1_ in the reflectance domain [44].

The similarity measure is used in two places in this workflow: extracting features from HSI and using them as kernels in the K-means clustering. The difference between these two usages is that extracting feature uses the mean value (*μ*) of the whole HSI as the competitor but using them as kernels in the K-means clustering uses the mean value of *j*th centroids (*μ^j^*) of the HSI as the competitor.

#### 2.2.2. Feature Selection

A feature selection technique aims to remove irrelevant or redundant features and keep relevant features for a dataset [26]. There are three ways to carry out the feature selection: filter, wrapper, and embedded [45]. In this study, filter feature selection was used for the classification method. The Pearson correlation was used to check the correlation between any of the two features,

(1) $$\rho =\frac{\sum \left(a_{i}-\bar{a}\right)\left(b_{i}-\bar{b}\right)}{\sqrt{\sum {\left(a_{i}-\bar{a}\right)}^{2}\sum {\left(b_{i}-\bar{b}\right)}^{2}}},$$

*a* and *b* represent the vector belonging to the different features. Then, the minimum of the absolute Pearson correlation ($\min\left(\left|\left(\rho_{1}\ldots \rho_{n}\right)\right|\right)$) was found. It represents that the features have the most noncorrelation, which means they have the least duplicated information. After finding the two similarity measures, a *CV* was used to compute the variation in each similarity measure, which is similar to ANOVA. If a feature has a large variation, that means this feature is easy to be recognized in this dataset. The equation to calculate *CV* is

(2) $$CV_{S}=\bar{\sum_{0}^{m}\frac{\sigma \left(S(X_{n},X_{n+1}\right))}{\mu \left(S(X_{n},X_{n+1}\right))}},$$

*CV_S_* is the *CV* for a similarity measure. $\sigma \left(S(X_{n},X_{n+1}\right))$ is the standard deviation of a similarity measure between two pixels (*X_n_* and *X_n_*_+1_). $\mu \left(S(X_{n},X_{n+1}\right))$ is the mean of a similarity measure between two pixels (*X_n_* and *X_n_*_+1_). While calculating *CVs* for different features, the ratio between each *CV* was also computed because the *CV* is dimensionless. The ratio was used as the weight of each similarity measure in the K-means clustering method in the next section. The ratio is calculated by one restriction as follows

(3) $$\{\begin{array}{c} \frac{CV_{S1}}{CV_{S2}}=\frac{w_{1}}{w_{2}}\\ w_{1}+w_{2}=1 \end{array},$$

where *CV_S_*_1_ and *CV_S_*_2_ represent the *CV* of two similarity measures and *w*_1_ and *w*_2_ are the weights of these two.

#### 2.2.3. Hybrid Classification Method

A hybrid classification method is a classification method that contains two or more kernels with different similarity measures [21,23]. In this paper, K-means was used as the clustering algorithm for the hybrid classification method [46,47]. The algorithm is

1. Specify the number clusters as *K*;
2. Randomly select *K* centroids among samples;
3. Keep iterating the following equations until no change to the centroids.

For one sample $x^{i}$, calculating the belonging cluster of this sample,

(4) $$C^{i}=\{\begin{array}{c} \arg\min\|X^{i}-\mu_{j}{\|}^{2}\\ 0,\mathrm{otherwise} \end{array},\;\mathrm{if}\;j\;\mathrm{belongs}\;\mathrm{to}\;\mathrm{the}\;\mathrm{cluster},$$

*j* is the *j*th centroids and it is [0, *K*]. *I* is the *i*th sample and it is [0, *the number of samples*]. *x^i^* represents a sample. *c^i^* represents a cluster that *x^i^* is the most likely belonging to.$\;\mu_{j}\;$represents the centroid of a cluster. arg *min* is an argument of the minimum, which means a function attains its minimum.

For one cluster $\mu_{j}$, recalculating the centroid of this cluster,

(5) $$\mu_{j}=\frac{1}{\left|c^{i}\right|}\sum x^{i},\;\;x^{i}\in c^{i}.$$

For the HCW-SSC method, the calculation of the belonging cluster of one point (Equation (4)) is changed to:

(6) $$\arg min\;||w_{1}\times \mathrm{similarity}\;\mathrm{measure}1+w_{2}\times \mathrm{similarity}\;\mathrm{measure}2|{|}^{2}\;$$

(7) $$w_{1}=\left(\frac{CV_{S1}}{CV_{S1}+CV_{S2}}\right)/\bar{\mu_{S1}},$$

(8) $$w_{2}=\left(\frac{CV_{S2}}{CV_{S1}+CV_{S2}}\right)/\bar{\mu_{S2}},$$

*w*_1_ and *w*_2_ were calculated from feature selection in the previous step. $\bar{\mu_{S}}$ and $\bar{\mu_{S+1}}$ are the means of similarity measures to remove the dimension. For example, ED and SAC were selected as the most useful features, the equation will be

(9) $$\arg min\;||w_{1}\sqrt{\|X_{i}-X_{j}{\|}^{2}}+w_{2}{\left(1-\frac{\sum X_{i}X_{j}}{\sqrt{\sum X_{i}{}^{2}}\sqrt{\sum X_{j}{}^{2}}}\right)||}^{2}.$$

### 2.3. Evaluation Indicators

For the test of standard spectral libraries, the overall accuracy and *F*1 score were used as the evaluation indicator [48]. For the tests of HSIs, the overall accuracy and kappa coefficient were used as the evaluation indicator [49,50]. In a confusion matrix, columns represent a true number of pixels in each class, and rows represent the predicted number of pixels in each class. The matrix is square and all numbers of the correct classified pixels are along the upper-left to lower-right diagonal. The overall accuracy is the ratio of the number of correctly classified pixels to the total number of pixels [2]. The equation is

(10) $$OverallAccuracy=\frac{the\;sum\;of\;the\;numbers\;on\;the\;diagonal}{Total},$$

Kappa is designed to compare the accuracy of a classification method to the accuracy of a random selection [48]. Kappa is dimensionless and the value is from −1 to 1. The equation is

(11) $$Kappa=\frac{totalAccuracy-randomAccuracy}{1-randomAccuracy},$$

(12) $$totalAccuracy=\frac{TP+TN}{Total},$$

(13) $$randomAccuracy=\frac{\left(TN+FP\right)\times \left(TN+FN\right)+\left(FN+TP\right)\times \left(FP+TP\right)}{Total\times Total}.$$

*TP* is true positive, which is the number of pixels in a given class that were classified correctly. *TN* is true negative, which is the number of pixels in a given class that were not classified correctly. *FP* is false positive, which is the number of pixels that were predicted to be in a given class but do not belong to that class. *FN* is false negative, which is the number of pixels that were not predicted to be in a given class but do belong to that class.

*F*1 score is designed to check the balance of precision and recall. The equation is

(14) $$F1=2\times \frac{Precision\times Recall}{Precision+Recall},$$

(15) $$Precision=\frac{TP}{TP+FP},$$

(16) $$Recall=\frac{TP}{TP+FN}.\;$$

### 2.4. Implementation

The number of clusters relies on the elbow method, which calculated distortion and inertia per number of clusters. For testing of standard spectral libraries, the number of clusters is 2; the maximum iteration of the K-means clustering method with ED, SAC, ED-SAC, and HCW-SSC similarity measure is 2; and the minimum change threshold is 2%. For testing of OMIS HSI, the number of clusters is 7; the maximum iteration of K-means clustering method with SAC, SID, SID-SAC, and HCW-SSC similarity measure is 6; and the minimum change threshold is 5%. For testing of AVIRIS HSI, the number of clusters is 5; the maximum iteration of K-means clustering method of ED, SAC, ED-SAC, and HCW-SSC similarity measure is 5; and the minimum change threshold is 5%. For testing of Hyperion HSI, the number of clusters is 11; the maximum iteration of K-means clustering method of SID, SCC, SID-SCC, and HCW-SSC similarity measure is 9; and the minimum change threshold is 5%. For random forest (RF) classification in both AVIRIS and Hyperion imagery, the number of trees in the forest is 100 and the maximum depth of a tree is 5 (the RF classifier was directly applied on the HSI, which was not applied on feature selection).

The preprocess of HSI was made by ENVI 5.3 (L3 Harris Technologies, Boulder, CO, USA). The codes of feature selection and hybrid classification were written in Python 3.8 with GDAL and scikit-learn packages [51,52].

## 3. Results

### 3.1. Test Based on Standard Spectral Libraries

ED and SAC were selected as the kernels of K-means in the HCW-SSC method. The single-kernel ED, single-kernel SAC, and the unweighted ED and SAC kernels were also implemented in the K-means as the comparisons. The HCW-SSC method resulted in the highest overall accuracy, followed by ED-SAC (Table 3). The single ED produced the lowest overall accuracy.

### 3.2. Test Based on OMIS HSI

SAC and SID were selected as the kernels of K-means in the HCW-SSC method. The single-kernel SAC, the single-kernel SID, and the unweighted SID-SAC kernels were also implemented in the K-means as the comparisons. The overall accuracy and kappa coefficient were calculated to assess the four types of spectral similarity measures (Table 4). The HCW-SSC was the highest, whether in overall accuracy or kappa coefficient. The overall accuracy was 93.21% and the kappa coefficient was 0.9245.

**Figure 6:**
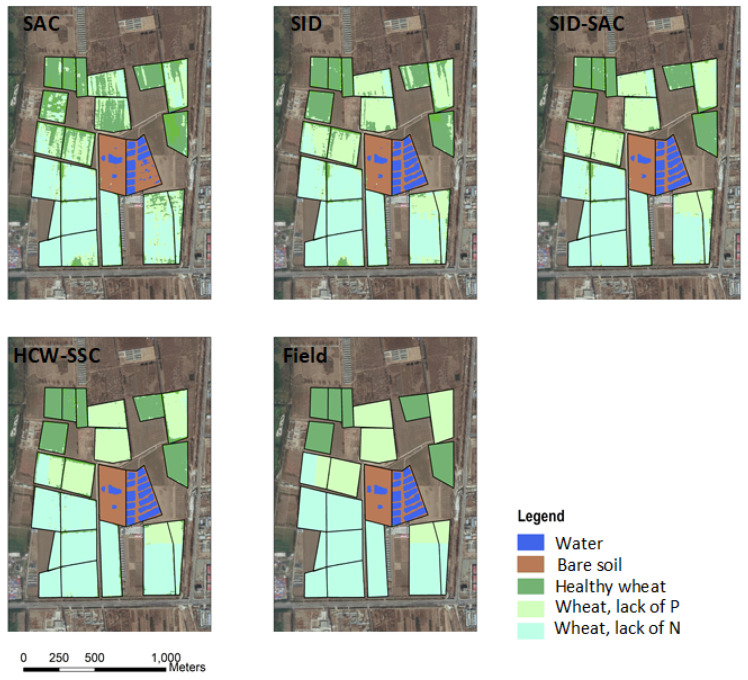
Classification maps of four different classification methods and the field measurement (spectral angle cosine (SAC), spectral information divergence (SID), spectral information divergence–spectral angle cosine (SID-SAC), and a new hybrid changing-weight classification method with a filter feature selection (HCW-SSC)).

Compared to the classification maps of field measurement, the HCW-SSC method produced the best classification result, followed by SID-SAC (Figure 6). Single SAC and SID cannot effectively classify several intercropping or interbreeding wheat areas, because the spectra of winter wheat in different colonies were similar. Therefore, only using a single similarity measure of spectral curves and spectral vectors cannot distinguish the winter wheat of different colonies. SID-SAC reflected a better classification effect as a whole, but an “island problem” about classification exists in some centralized winter wheat areas. It did not comply with the actual growing conditions of the wheat. The HCW-SSC method has the less-misclassified pixels and the cleanest boundary.

### 3.3. Test Based on AVIRIS HSI

ED and SAC were selected as the kernels of K-means in the HCW-SSC method. The single kernel ED, the single kernel SAC, and the unweighted ED-SAC kernels were also implemented in the K-means as the comparisons. The total classification accuracy and kappa coefficient were calculated to assess the four types of spectral similarity measures (Table 5). The HCW-SSC was the highest, whether in overall accuracy or kappa coefficient. Overall accuracy was 79.24% and the kappa coefficient was 0.8044.

**Figure 7:**
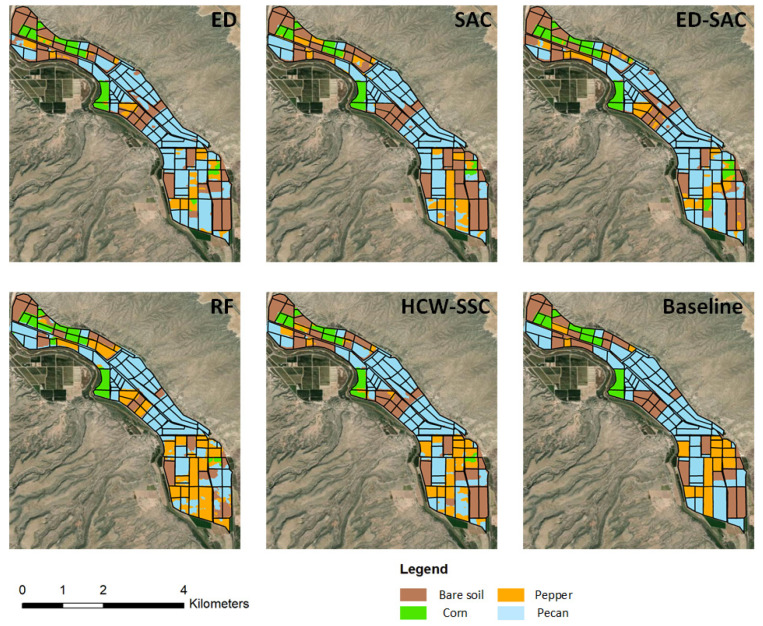
Classification maps of five different classification methods and baseline map (Euclidean distance (ED), spectral angle cosine (SAC), Euclidean distance–spectral angle cosine (ED-SAC), random forest (RF), and a new hybrid changing-weight classification method with a filter feature selection (HCW-SSC)).

Compared to the baseline classification maps, the HCW-SSC method produced the best classification result, followed by ED-SAC (Figure 7). Single ED or SAC has misclassified some land covers such as pepper and pecan, especially on the mixing area of pepper and pecan. RF has less misclassification since it is an ensemble model, which can select the best solution among all solutions.

### 3.4. Test Based on Hyperion HSI

SID and SCC were selected as the kernels of K-means in the HCW-SSC method. The single-kernel SID, the single-kernel SCC, and the unweighted SID-SCC kernels were also implemented in the K-means as the comparisons. The total classification accuracy and kappa coefficient were calculated to assess the four types of spectral similarity measures (Table 6). The HCW-SSC was the highest, whether in overall accuracy or kappa coefficient. Overall accuracy was 81.23% and kappa coefficient was 0.7234.

**Figure 8:**
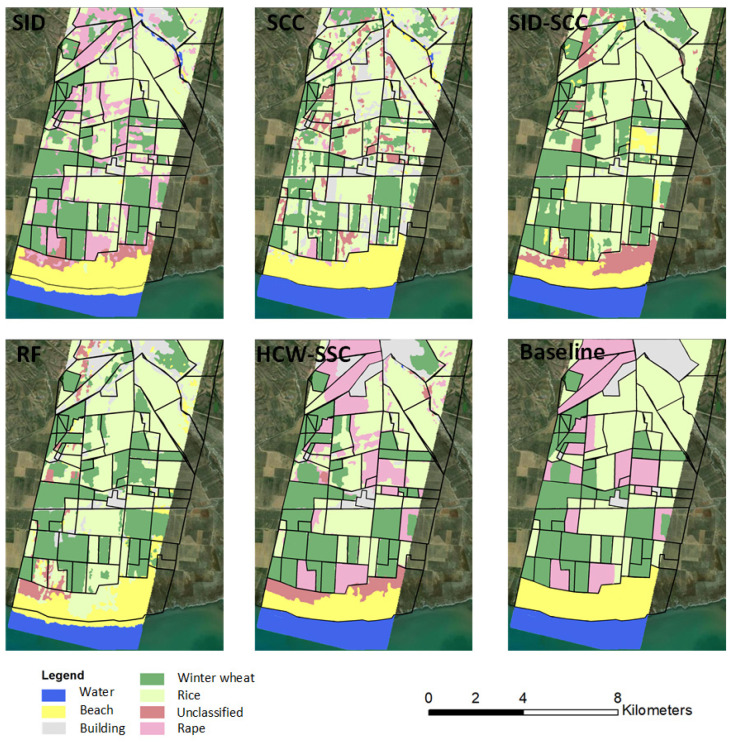
Classification maps of five different classification methods and baseline map (spectral information divergence (SID), spectral correlation coefficient (SCC), spectral information divergence–spectral correlation coefficient (SID-SCC), random forest (RF), and a new hybrid changing-weight classification method with a filter feature selection (HCW-SSC)).

Compared to the baseline classification maps, the HCW-SSC method produced the best classification result, followed by SCC (Figure 8). Single SID or SCC has an obvious “island problem”, which means the spectrum of the adjunct pixel surrounding the “island” is similar and it is hard to use only one similarity measure to classify. RF and SID-SCC almost cannot classify the rape from winter wheat and rice, which was caused by the overfitting by the algorithm.

## 4. Discussion

In this paper, the HCW-SSC method only chose features from four basic similarity measures and the weight restriction is naive. In the future, more similarity measures can be used to extract the features from HSI and then be implemented into K-means as the kernels. In this section, the importance of feature selection in HCW-SSC and the performance of HCW-SSC are discussed.

### 4.1. The Importance of Feature Selection in HCW-SSC

#### 4.1.1. Select the Suitable Similarity Measures as Kernels

HCW-SSC was compared to the unweighted hybrid kernel that is composed of selected similarity measures from filter feature selection. However, the classification results of these hybrid kernels that are composed of non-selected similarity measures were not shown. In Table 7, these classification results are shown. The unweighted SID-SAC has the highest accuracy among all unweighted hybrid kernels (ED-SCC, ED-SAC, SCC-SID, SAC-SCC, and ED-SID). That means if two or more similarity measures were randomly implemented into a kernel, the classification result of it may not become more accurate. Therefore, choosing appropriate kernels by feature selection for a hybrid classification method is important.

#### 4.1.2. Calculate the Weights for the Kernels

The unweighted hybrid method directly multiplies two similarity features. A different similarity measure has a different unit, so it led to the unbalanced usage of the two kernels. HCW-SSC took the weight from CV and used CV divided by mean to make the similarity measure unitless. The weight helps balance the contribution of two similarity measures for the K-means clustering. Therefore, the hybrid classification method (HCW-SSC) with a feature selection technique generates a better result than the one without it. The weights of the HCW-SSC method based on the standard spectral libraries are shown in Table 8. The mean weight of Euclidean distance fluctuated at 0.31, whereas the mean weight of spectral angle cosine fluctuated at 0.69. SAC contributes more than ED in this dataset for the classification. However, in the ED-SAC method, the absolute value of ED is much larger than SAC, so ED takes more contribution than SAC, which leads to more misclassifications.

### 4.2. The Time Complexity of HCW-SSC Method

**Figure 9:**
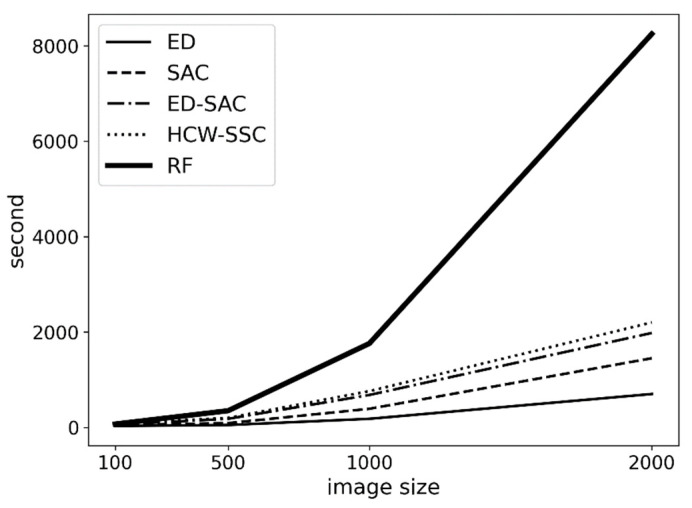
The time consumption of different kernels is based on different image sizes (Euclidean distance (ED), spectral angle cosine (SAC), Euclidean distance–spectral angle cosine (ED-SAC), a new hybrid changing-weight classification method with a filter feature selection (HCW-SSC), and random forest (RF)).

Another advantage of this method is its performance. Based on Figure 9, HCW-SSC has a similar time consumption to the traditional method (ED-SAC). Although HCW-SSC has the filter feature selection, the filter feature selection takes linear time to visit all pixels; therefore, the total time complexity is still linear (O(n), the operation of multiply and sum has linear time complexity). However, the time complexity of a random forest is n-square (O(n^2^)) because each new tree in the forest will bring n more leaves. When the image becomes larger, the time consumption of random forest becomes much larger.

### 4.3. Compared to Other Methods

There are three research topics for the classification method of HSIs: clustering based on graph theory [16,17], clustering by using a machine-learning algorithm [18,19], and clustering with the hybrid kernels [21,22,23,24]. The HCW-SSC represents the clustering with the hybrid kernels. Compared to the non-filter-selection method, the new method performs much better (Table 3, Table 4, Table 5 and Table 6). In order to compare the method of clustering based on graph theory and clustering by using a machine-learning algorithm, three papers were selected. Although the HSI data in these three papers are different from this paper, they indirectly show the accuracy of the HCW-SSC method.

In the paper by Meng et al., 2017 [16], they used a semisupervised kernel based on graph theory with K-means clustering to classify three HSIs. The result is in Table 9. For AVIRIS and airborne sensor HSIs, the HCW-SSC method has a similar overall accuracy (79.24% and 93.21%) with this semisupervised kernel (80.37% and 99.17%). The semisupervised kernel is based on the graph theory, which highly depends on the performance of the GPU and the storage of RAM. However, the HCW-SSC method does not need GPU and extra RAM.

In the paper by Li et al., 2019 [20], they used four machine-learning algorithms and six convolutional neural network (CNN)-related deep-learning algorithms to classify three HSIs. The result is in Table 10. For AVIRIS and airborne sensor HSIs, the HCW-SSC method has a similar overall accuracy (79.24% and 93.21%) to the mean overall accuracy (78.79% and 95.80%) of the ten methods in Li’s paper. The HCW-SSC has linear time complexity but the machine-learning method, especially the deep-learning method, has polynomial or exponential time complexity.

In the paper by Yuan et al., 2016 [25], they used four machine-learning algorithms to classify three HSIs. The result is in Table 11. For AVIRIS HSIs, the HCW-SSC method has a similar overall accuracy (79.24%) to the mean overall accuracy (74.94%) of the four methods in Yuan’s paper.

## 5. Conclusions

This paper raised a hybrid classification method that can utilize the features chosen from filter feature selection on hyperspectral images, which is called HCW-SSC. Standard spectral libraries, OMIS airborne hyperspectral image, AVIRIS hyperspectral image, and Hyperion satellite hyperspectral image were used to inspect the accuracy of the HCW-SSC classification method. The results showed that the HCW-SSC method has the highest overall accuracy and kappa coefficient (or F1 score) in all experiments (97.5% and 0.974 for standard spectral libraries, 93.21% and 0.9245 for OMIS, 79.24% and 0.8044 for AVIRIS, and 81.23% and 0.7234 for Hyperion). The HCW-SSC method exhibits a better spectral-recognition capacity compared to the classification method with one type of spectral characteristic or simply combines two spectral characteristics and a machine-learning method. Compared to the other two research directions of HSI classification, HCW-SSC has a balance of time complexity and space usage but obtains similar accuracy. This paper can be a useful reference for how feature selection optimizes the traditional hybrid classification methods and improves the accuracy of classification methods for hyperspectral remote-sensing data. This also sheds a light on the importance of feature selection in HSI classification. In the future, this method may be applied to time-series remote-sensing data, which has the time-series curves of land cover rather than spectral curves.

## Figures and Tables

**Table 1:** The details of data used in this paper.

Name	Spatial Resolution	Spectral Resolution	Spectral Domain	Location (Based on WGS 84)
Standard spectral libraries	NA (fieldwork)	0.2 nm or 1 nm	400–2500 nm	Pasadena, CA, USA
OMIS	2.8 m	10 nm	400–12,500 nm	Beijing, China (40°10′57″ N, 116°26′36″ E)
AVIRIS	20 m	10 nm	357–2576 nm	NM, USA (32°28′16.6″ N, 106°54′23.7″ W)
Hyperion	30 m	10 nm	426–2356 nm	Qianhai, China (37°25′54″ N, 100°11′44″ E)

**Table 2:** The details of spectral libraries.

Spectral Library	The Extent of Wavelength	Spectral Resolution in the Visible Region	Spectral Resolution in the Infrared Region
USGS vegetation	0.4–2.5 μm	0.2 nm	0.5 nm
USGS mineral	0.4–2.5 μm	0.2 nm	0.5 nm
Chris Elvidge green	0.4–2.5 μm	1 nm	4 nm
Chris Elvidge dry	0.4–2.5 μm	1 nm	4 nm

**Table 3:** Overall accuracy of each similarity measure based on standard spectral libraries (Euclidean distance (ED), spectral angle cosine (SAC), Euclidean distance–spectral angle cosine (ED-SAC), and a new hybrid changing-weight classification method with a filter feature selection (HCW-SSC)).

Similarity Measure	ED	SAC	ED-SAC	HCW-SSC
Overall accuracy (%)	87.50	91.25	93.75	97.50
F1 score	0.888	0.943	0.958	0.974

**Table 4:** Overall accuracy and kappa coefficient of each similarity measure based on OMIS HSI (spectral angle cosine (SAC), spectral information divergence (SID), spectral information divergence–spectral angle cosine (SID-SAC), and a new hybrid changing-weight classification method with a filter feature selection (HCW-SSC)).

Similarity Measure	SAC	SID	SID-SAC	HCW-SSC
Overall accuracy (%)	62.69	75.69	88.27	93.21
Kappa coefficient	0.5989	0.7563	0.8698	0.9245

**Table 5:** Overall accuracy and kappa coefficient of each similarity measure based on AVIRIS HSI (Euclidean distance (ED), spectral angle cosine (SAC), Euclidean distance–spectral angle cosine (ED-SAC), random forest (RF), and a new hybrid changing-weight classification method with a filter feature selection (HCW-SSC)).

Similarity Measure	ED	SAC	ED-SAC	RF	HCW-SSC
Overall accuracy (%)	51.23	63.76	73.12	68.27	79.24
Kappa coefficient	0.4249	0.5323	0.7225	0.6628	0.8044

**Table 6:** Overall accuracy and kappa coefficient of each similarity measure based on Hyperion HSI (spectral information divergence (SID), spectral correlation coefficient (SCC), spectral information divergence–spectral correlation coefficient (SID-SCC), random forest (RF), and a new hybrid changing-weight classification method with a filter feature selection (HCW-SSC)).

Similarity Measure	SID	SCC	SID-SCC	RF	HCW-SSC
Overall accuracy (%)	54.82	62.39	56.34	51.21	81.23
Kappa coefficient	0.3934	0.4363	0.3623	0.4255	0.7234

**Table 7:** Overall accuracy and kappa coefficient of each hybrid kernel based on OMIS HSI (the gray area has the results from Table 4, Euclidean distance–spectral correlation coefficient (ED-SCC), Euclidean distance–spectral angle cosine (ED-SAC), spectral correlation coefficient–spectral information divergence (SCC-SID), spectral angle cosine–spectral correlation coefficient (SAC-SCC), Euclidean distance–spectral information divergence (ED-SID), spectral information divergence–spectral angle cosine (SID-SAC), and a new hybrid changing-weight classification method with a filter feature selection (HCW-SSC)). The numbers in bold mean the most important results.

Similarity Measure	ED-SCC	ED-SAC	SCC-SID	SAC-SCC	ED-SID	SID-SAC	HCW-SSC
Overall accuracy (%)	63.22	58.82	61.39	69.24	66.17	88.27	**93.21**
Kappa coefficient	0.6215	0.4932	0.4342	0.7014	0.6138	0.8698	**0.9245**

**Table 8:** Weight of selected similarity measures based on standard spectral libraries (Euclidean distance (ED) and spectral angle cosine (SAC)).

Weight	Types of the Standard Spectral Library			
	USGS Vegetation	Chris Elvidge Dry	Chris Elvidge Green	USGS Mineral
ED	0.312	0.329	0.343	0.246
SAC	0.688	0.671	0.657	0.754

**Table 9:** Overall accuracy of a semisupervised kernel with K-means on three different datasets.

	Urban (Hypercube)	Kennedy Space Center (AVIRIS)	DC Mall (Airborne Sensor)
Number of bands	210	176	210
Number of classes	4	13	7
Overall Accuracy			
Semisupervised kernel	93.48%	80.37%	99.17%

**Table 10:** Overall accuracy of support vector machines (SVM), extended morphological profiles (EMPs), joint spare representation (JSR), edge-preserving filtering (EPF), 3D-CNN, CNN with pixel–pair features (CNN-PPF), Gabor-CNN, Siamese CNN (S-CNN), 3D-generative adversarial network (3D-GAN), and the deep feature fusion network (DFFN) on three different datasets.

	Salinas (AVIRIS)	University of Pavia (ROSIS-03 Sensor)	Houston (Airborne Sensor)
Number of bands	204	115	144
Number of classes	16	9	15
Overall Accuracy			
SVM	0.7513	0.8813	0.8985
EMP	0.7815	0.9504	0.9707
JSR	0.7528	0.9349	0.9307
EPF	0.7824	0.9688	0.9700
3D-CNN	0.8013	0.9502	0.9695
CNN-PPE	0.7991	0.969	0.9404
Gabor-CNN	0.8114	0.9662	0.9734
S-CNN	0.8052	0.9743	0.9510
3D-GAN	0.7616	0.9697	0.9793
DFFN	0.8328	0.9808	0.9967

**Table 11:** Overall accuracy of support vector machines (SVM), k-nearest neighbors (kNN), classification and regression trees (Cart), and naïve Bayes on three different datasets.

	Indian Pines (AVIRIS)	Salinas (AVIRIS)	University of Pavia (ROSIS-03 Sensor)
Number of bands	224	224	103
Number of classes	9	16	9
Overall Accuracy			
SVM	0.7875	0.8994	0.8992
kNN	0.6733	0.8502	0.7942
Cart	0.6301	0.8389	0.7406
Naïve Bayes	0.5292	0.7873	0.6776

## Data Availability

United States Geological Survey (USGS) vegetation and mineral libraries can be found in Clark et al., 1993 and Chris Elvidge green and dry vegetation libraries can be found in Elvidge 1990. The OMIS data can be found at http://www.scidb.cn/en (accessed on 8 June 2012). The AVIRIS data can be found at https://aviris.jpl.nasa.gov/dataportal/ (accessed on 9 May 2015). The EO-1 data can be found at https://www.planet.com/products/planet-imagery/ (accessed on 9 February 2015). NAIP data can be found at https://nrcs.app.box.com/v/naip (accessed on 9 May 2015). Rapideye data can be found at https://www.planet.com/products/planet-imagery/ (accessed on 9 May 2015).
